# The Springfield Meeting

**Published:** 1883-06

**Authors:** 


### THE SPRINGFIELD MEETING.

The union meeting of the Massachusetts State and the Connecticut Valley Dental Associations was an eminently successful one. Whether we consider the number in attendance, the enthusiasm manifested, the character and intelligence of those present, the value and importance of the papers read, or the earnestness of the discussions, from almost any standpoint, in fact, the meeting was a memorable one. It was admirably managed, too, and there was scarce one unpleasant incident connected with it.

The executive committees had arranged for the thorough presentation of two subjects which are now specially interesting the profession—the etiology of dental caries, and artificial crowns. In etiology, nearly all the modern theories were presented by able essayists, and the discussion over them was earnest and exhaustive.

Drs. Bonwill, Buttner, Parmly Brown, and H. A. Baker gave demonstrations of the various methods of attaching artificial crowns, and Dr. Bliven illustrated the method of making dental instruments. There were a number of papers read upon other subjects, but space will not permit our enlarging upon them. Concerning the knowledge and enthusiasm manifested, it is sufficient to say that a body of dentists who, with scarce an exception, will, upon one of the most oppressive days of summer, listen anxiously for nearly three hours to one speaker while he labors to unfold a technical and obscure subject, must be exceptionally intelligent.

Our western friend, Dr. J. A. Robinson, of Michigan, was there, claiming, as usual, kinship with every one present. He read a capital Robinsonian paper, too, which we expect to present to our readers in the near future.

We regret that we cannot present a full report of the papers and discussions, but Springfield has an excellent dental journal of its own, and to that we must refer any of our readers who desire a more particular account of this interesting meeting.

